# ELISA-like Analysis of Cisplatinated DNA Using Magnetic Separation

**DOI:** 10.5772/61699

**Published:** 2015-01-01

**Authors:** Kristyna Smerkova, Marcela Vlcnovska, Simona Dostalova, Vedran Milosavljevic, Pavel Kopel, Tomas Vaculovic, Sona Krizkova, Marketa Vaculovicova, Vojtech Adam, Rene Kizek

**Affiliations:** 1 Department of Chemistry and Biochemistry, Laboratory of Metallomics and Nanotechnology, Mendel University in Brno, Brno, Czech Republic; 2 Central European Institute of Technology, Brno University of Technology, Brno, Czech Republic; 3 Department of Chemistry, Faculty of Science, Masaryk University, Brno, Czech Republic

**Keywords:** Anti-DNA Antibodies, Cisplatin, Magnetic Separation, Sandwich Analysis

## Abstract

Cisplatin belongs to the most widely used cytostatic drugs. The determination of the presence of the DNA-cisplatin adducts may not only signal the guanine-rich regions but also monitor the interaction reaction between DNA and the drug in terms of speed of interaction.

In this work, the combined advantages of magnetic particles-based isolation/purification with fluorescent properties of quantum dots (QDs) and antibodies targeted on specific recognition of DNA-cisplatin adducts are demonstrated. The formation of a complex between magnetic particles with surface modified by anti-dsDNA antibody, cisplatin-modified DNA and QDs labelled anti-cisplatin-modified DNA antibody was suggested and optimized.

## 1. Introduction

The method of magnetic separation can be employed in the analysis of many different compounds, from ions [1–3] and amino acids [4–6] through nucleic acids [7–9] and proteins [10–12] to virions [13–15] or even whole bacterial cells [16–18]. Spherical structures of nanometric or micrometric size with paramagnetic or superparamagnetic properties are used in magnetic separation [6].

The surface of magnetic particles (MPs) is modified either with a charged layer for electrostatic adsorption or ligands for specific bond with biomolecules [19]. One of the frequently used ligands is protein G, naturally occurring in Streptococcal bacteria. It is a cell surface protein with the ability to bind immunoglobulins via their Fc region. Thus, the antibodies retain their immunoprecipitating activity even after binding to MPs [20]. A wide range of both monoclonal and polyclonal antibodies can be bound to protein G, including human, mouse, rat, bovine, goat or rabbit [21].

The antibodies bound on MPs can subsequently be used in an ELISA-like method for identification or analysis of a desired antigen [12]. The secondary antibody for sandwich ELISA can be labelled according to standard protocols either with enzyme or fluorescent nanoparticles [22]. Use of micro- and nanoparticles in immunoassays allows shortened analysis time with minimal sample requirement and higher sensitivity compared to common formats [23, 24]. In this work, we employed the sandwich ELISA-like method using magnetic separation for selective analysis of DNA modified with the cytotoxic drug cisplatin. The affinity of anti-dsDNA primary and anti-cisplatin-modified DNA secondary antibodies to DNA was confirmed with a dot blot technique. The secondary antibody was labelled with QDs for fluorescent detection of DNA.

## 2. Materials and Methods

### 2.1 Chemicals

All chemicals of ACS purity were obtained from Sigma-Aldrich (St. Louis, MO, USA) unless otherwise stated. Deionized water underwent demineralization by reverse osmosis using the instrument Aqua Osmotic 02 (Aqua Osmotic, Tisnov, Czech Republic), followed by further purification using Millipore RG (Millipore, Billerica, MA, USA) Milli-Q water. The pH was measured using a WTW inoLab pH meter (Weilheim, Germany).

### 2.2 Amplification of DNA fragments by polymerase chain reaction

DNA isolated from bacteriophage λ (48 502 bp) and a *Taq* PCR kit were purchased from New England Biolabs (Ipswich, MA, USA). Primers were synthesized by Sigma-Aldrich (St. Louis, MO, USA) and their sequence was 5′-CCTGCTCTGCCGCTTCACGC-3′ for forward primer and 5′-TCCGGATAAAAACGTCGATGACATTTGC-3′ for reverse primer. The volume of reaction mixture was 50μL and it was composed of 5μl of 10× standard *Taq* reaction buffer; 1μL of 1mM deoxynucleotide solution; 1μL of each 10μM primer; 0.25μL of *Taq* DNA polymerase; 40.75μL of water (sterile) and 1μL of 0.5μg/μL DNA. PCR took place in Mastercycler ep *realplex*^4^ S (Eppendorf, Hamburg, Germany) and the cycling conditions were as follows: denaturation for 120 s at 95 °C; 30 cycles of denaturation for 15 s at 95 °C, annealing for 15 s at 64 °C and elongation for 45 s at 72 °C with a final elongation for 5 min at 72 °C. Obtained DNA fragments (498 bp) were purified using the MinElute PCR Purification Kit (Qiagen, Hilden, Germany). DNA concentration was determined spectrophotometrically (Analytic Jena, Jena, Germany).

### 2.3 Cisplatin interactions with DNA

DNA fragments solution (final concentration of 40.62nM) was mixed with cisplatin (final concentration of 0.13, 0.26, 0.52, 1.04, 2.08, 4.17, 8.33 and 16.66μM) in the environment of 10mM NaClO_4_. Solutions of DNA with drugs were incubated for 24 hours at 37 °C.

### 2.4 Dot blot assay

2μL of the DNA samples (0.1 – 162.5nM DNA fragment or 40.6nM DNA fragment with 0.1 – 16.7μM cisplatin) were immobilized on a Zeta Probe membrane (Bio-Rad, Hercules, CA, USA) and dried at 37 °C in an incubator Galaxy 14S (Eppendorf, Hamburg, Germany). The membrane was blocked for 30 min during rotation at 40 RPM (Multi RS-60, Biosan, Latvia) in a blocking buffer containing 1% milk powder in PBS (137mM NaCl, 2.7mM KCl, 1.4mM NaH_2_PO_4_ and 4.3mM Na_2_HPO_4_, pH 7.4). The solution was replaced by an antibody dilution buffer (1mg/mL bovine serum albumin in PBS) with the primary antibodies (anti-dsDNA antibody or anti-cisplatin modified DNA antibody, Abcam, Cambridge, England). The primary antibodies were diluted by buffer in the ratio 1:1000. The membrane was incubated with antibodies for 60 minutes at room temperature during rotation and then washed with PBS with 0.05% Tween 20 (PBS-T). The secondary polyclonal rabbit anti-mouse antibodies labelled with horseradish peroxidase (Dako, Denmark) in dilution of 1:1500 in dilution buffer were added to the membrane and incubated for 60 minutes during rotation. Visualization in chromogenic substrate followed after washing with PBS-T. The membrane was immersed in the solution composed of substrate buffer (0.5M acetate buffer, pH 5.4), 0.4mg/mL 3-amino-9-ethylcarbazole and hydrogen peroxide in the ratio 1000:10:1. The assay was performed according to [25].

The mean intensity of the colour was quantified using the Carestream Molecular Software (Rochester, NY, USA) in each spot and the colour of the background was deducted.

### 2.5 CdTe quantum dots preparation

Under magnetic stirring 1mL of aqueous solution of Cd(OAc)_2_ (5.32mg/mL) was added to 7.6mL of Milli-Q water. Then 1mg of HWRGWVC heptapeptide (abbreviated HWR peptide) and 50μL of 60mg/mL mercaptosuccinic acid were added. Preparation proceeded by adding 90μL of 1M NH_3_ and 150μl of 4.43mg/mL Na_2_TeO_3_. Reductions of samples were conducted by adding 4mg of NaBH_4_. Solutions were closed in vials and added into the Multiwave 3000Microwave Reaction System (Anton Paar, Graz, Austria) for 20 min at 110 °C and power of 300 W. To remove the excess HWR peptide, the QDs were filtered by the Amicon Ultra 3K device (Millipore, Billerica, MA, USA).

### 2.6 The magnetic particles-based immunoassay

The magnetic microparticles Dynabeads Protein G (Life Technologies, Carlsbadt, CA, USA) and magnetic separation rack MagnaRack (Life Technologies, Carlsbad, CA, USA) were used for immunoseparation. The anti-dsDNA antibodies' immobilization on the MPs' surface was done according to the manufacturer's recommendations. The storage solution was removed from the 50μL (1.5mg) of resuspended MPs in a tube on the magnetic rack. 10μg of anti-dsDNA antibodies in 200μL of PBS-T was added to the MPs. In the case of binding capacity optimization the 0.25, 0.5, 1, 2, 5, 8, 10 and 15μg of anti-dsDNA antibodies were added to 1mg of MPs. The MPs were incubated with antibodies for 30 min during rotation at 40 RPM and room temperature. After that, the tube with the MPs was placed on the magnetic rack, the supernatant was removed and the MPs-antibodies complex was washed by 200μL of PBS-T. The MPs were resuspended in 200μl of PBS-T and 50μL (0.38mg) of this mixture was placed on the magnetic rack. The supernatant was removed and 10μL of DNA sample in 20μL of Tris-HCl (pH 8) with 0.1M NaCl was added. The DNA samples were 162.48nM PCR fragment or 40.62nM PCR fragment with 8.3 and 16.7μM cisplatin. The incubation with the MPs-antibodies complex was carried out for 60 min during rotation at 40 RPM and room temperature.

In the case of testing DNA binding to the MPs, the MPs were twice washed by 200μL of Tris-HCl (pH 8) with 0.1M NaCl. After the removal of the supernatant the washed MPs were incubated with 10μL of acetate buffer (0.2M sodium acetate and 1M NaCl, pH 4) for 60 min during rotation at 40 RPM and room temperature. Then the tube with MPs was placed on magnetic rack and the eluted DNA was removed to a new tube. The dialysis by 25nM membrane filter (Millipore, Billerica, MA, USA) for 60 min at 6 °C was used to remove the excess salt. The DNA samples were detected by agarose gel electrophoresis.

In the case of sandwich immunoassay, the MPs-antibodies complex with cisplatinated DNA was twice washed by 200μL of PBS-T. The 18μl of PBS-T and 2μL of anti-cisplatin-modified DNA antibody (1.47mg/mL) were added to the washed MPs with removed supernatant and the incubation was performed for 60 min during rotation at room temperature. After that, the immunosandwich on the MPs was twice washed with 30μL of water. Then the MPs were resuspended in water (10μL), the 10μL of CdTe QDs modified by HWR peptide were added and the solution was incubated for 60 min during rotation at 40 RPM and room temperature. After the transfer of the tube with the MPs onto a magnetic rack, the MPs were twice washed with water (30μL). The washed MPs were resuspended in 50μL of water and the fluorescence of the coupled QDs was measured by the multifunctional microplate reader Tecan Infinite 200 PRO 132 (Tecan, Männendorf, Switzerland). The excitation wavelength was 360nM and the emission wavelength ranged from 390 to 850nM per 2nM steps.

### 2.7 Sodium dodecyl sulphate polyacrylamide gel electrophoresis (SDS-PAGE) of proteins

Each sample of MPs modified by anti-dsDNA antibody was diluted four times and then mixed with protein loading buffer (PLB) (under reducing conditions PLB with 3% mercaptoethanol) in a ratio of 2:1 and placed in the wells of the 12.5% polyacrylamide gel (*w/w*) prepared from 30% acrylamide/bis-acrylamide solution (37.5:1). Electrophoresis ran in 1× tris–glycine–SDS running buffer (3.02 g of Tris, 14.4 g of glycine, 1 g of SDS and water to a final volume of 1 L) for 70 min at a voltage of 120 V in the electrophoretic bath (Bio-Rad, Hercules, CA, USA). After that, the gels were visualized by silver staining.

### 2.8 Agarose gel electrophoresis

The DNA samples eluted from the MPs were analysed via agarose gel electrophoresis and the conditions were as follows: 1% agarose gel (Mercury, USA) with 1 × TAE buffer (40mM Tris, 20mM acetic acid and 1mM ethylenediamine-tetraacetic acid) and ethidium bromide (5μL/100mL of the gel), 100 V and 60 min (Bio-Rad, Hercules, CA, USA). The samples, prepared with 5% (*v/v*) bromophenol blue and 3% (*v/v*) glycerol, were loaded into a gel in 5μL aliquots. A 100 bp DNA ladder (New England BioLabs, Ipswich, MA, USA) within the size range from 0.1 to 1.5 kb was used to monitor the size of the analysed fragment. The bands were visualized via UV transilluminator at 312nM (Vilber-Lourmat, Marne-la-Vallée Cedex, France).

The ethidium bromide fluorescence intensity was evaluated using Carestream Molecular Software (Rochester, NY, USA). The mean intensity and maximum intensity of the fluorescence was quantified by software in each band and the fluorescence of the background was deducted.

### 2.9 Laser ablation-inductively coupled plasma mass spectrometry (LA-ICP-MS)

The LA-ICP-MS system, consisting of the laser ablation (LA) system UP213 (ESI, Portland, OR, USA) and the inductively coupled plasma mass spectrometer (ICP-MS) Agilent 7500ce (Agilent, Santa Clara, CA, USA), was used for quantitative determination of Pt in dot blots and in the sandwich immunocomplexes. The LA system serves for production of aerosol from the sample surface. Ablated aerosol is transported by carrier gas (helium with flow of 1.0 L/min) into ICP-MS.

For calibration purposes 2μL of solution containing Pt with concentration range 4 – 4000 ng/mL were deposited on the membrane surface. The formed spot was ablated by focused laser beam. The ablation was performed across the spot with laser beam diameter of 110μm, laser beam fluency of 5 J/cm^2^, repetition rate of 10 Hz and scan speed of 200μm/s. The ICP-MS signal of Pt was monitored using m/z = 195. The spots of the samples were analysed under the same LA-ICP-MS parameters.

## 3. Results and Discussion

**Figure 1.**
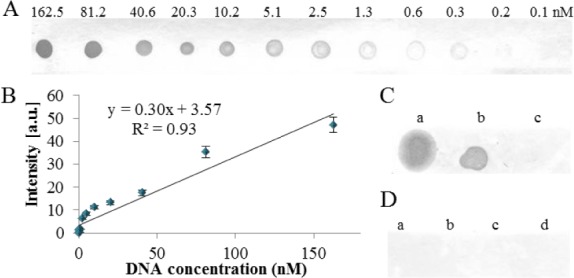
The anti-dsDNA antibody characterization by dot blot. (A) The calibration range of DNA fragment (498 bp) and (B) Determined spot intensities. (C) The dot blot of (a) human genomic DNA, (b) λ genomic DNA and (c) Human mRNA. (D) The dot blot of ss and ds oligonucleotides (ODN) at different lengths, (a) ssODN 43 bp, (b) dsODN 43 bp, (c) ssODN 17 bp and (d) dsODN 17 bp.

First, the reactivity of the anti-dsDNA and anti-cisplatin-modified DNA antibodies was tested by dot blot. In the case of the anti-dsDNA antibody, it was possible to detect 0.2nM concentration (0.1 ng) of a PCR product in a spot (Figure 1A, for details see *Materials and Methods* section). After determination of the spot's intensity, a linear response in the range of 0.2 – 162.5nM (0.1 – 100 ng) was obtained (Figure 1B). The reactivity of the antibodies to various types of nucleic acids was compared in the next step. The dsDNA (PCR product and human genomic DNA), total mRNA (derived human leucocytes), single-stranded (ss) oligonucleotides in length of 17 and 43 bp, ds oligonucleotides in length of 17 and 43 bp were spotted onto a membrane and immunodetected. From Figure 1C and 1D it is obvious that long dsDNA (e. g., PCR product and genomic DNA) exhibited immunoreactivity with anti-dsDNA antibodies only, while in the case of mRNA and both ss and ds oligonucleotides, no immunoreactivity was observed.

**Figure 2.**
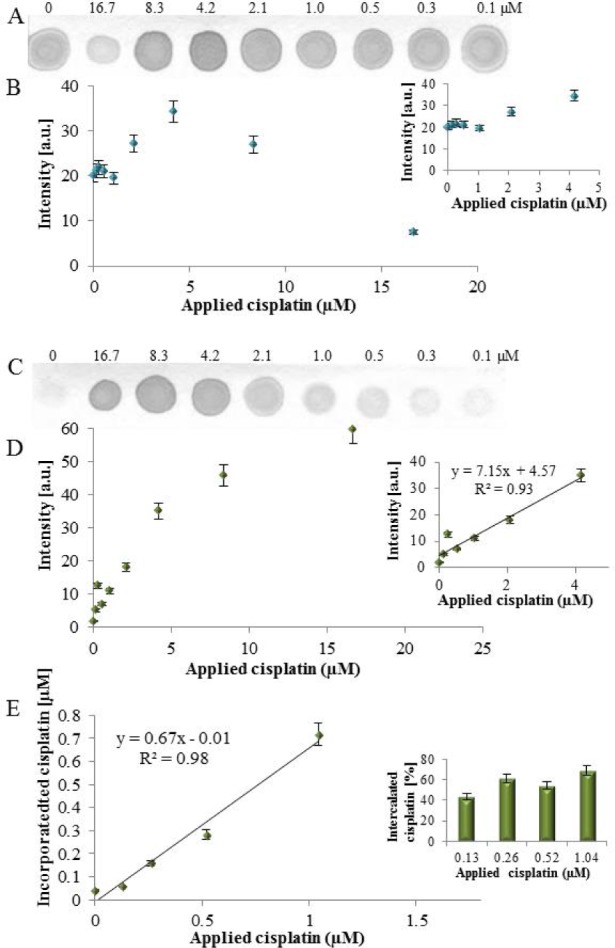
The cisplatinated DNA fragment detection by dot blot. To the DNA fragment (40.6nM) different cisplatin concentrations (0 – 16.7μM) were applied. (A) Interaction with anti-dsDNA antibody and (B) The determined spot intensities (inset: detailed view to applied cisplatin range 0 – 4.2μM). (C) Interaction with anti-cisplatin-modified DNA antibody and (D) The determined spot intensities (inset: the linear range). (E) The determination of cisplatin, incorporated within DNA, in spots by LA-ICP MS, (n = 3).

For verifying the immunoreactivity of the anti-cisplatinated DNA antibody the PCR product (40.6nM) was left to interact with different cisplatin concentrations (0 – 16.7μM). At all cisplatin concentrations the immunoreactivity with the anti-dsDNA antibody was recorded (Figure 2A), but contrary to expectations, the spot intensities varied in dependence on cisplatin concentration. A slight increase in the spot intensity in the range of 0 – 4.2μM concentrations (Figure 2B inset) was observed, while at higher cisplatin concentrations the spot's intensities decreased markedly (Figure 2B). This can be explained by cisplatin binding to the DNA bulk and affecting its structure, which is essential for immunorecognition, especially by formation of adducts, single strand and double strand breaks, guanine dimers and oxidative damage [26].

When the cisplatin-modified dsDNA reacted with the anti-cisplatin-modified DNA antibody, a linear response of the spot intensity in dependence on cisplatin concentration was obtained (Figure 2C and Figure 2D). This indicates that the antibody recognizes the structural changes of DNA caused by cisplatin binding. The marked discrepancy between the spot intensities at 8.3 and 16.7μM cisplatin concentrations obtained for anti-dsDNA and anti-cisplatin-modified DNA (see above) can be explained by the destruction of dsDNA structures essential for immunorecognition. The presence of Pt in the spots was verified by LA-ICP-MS and recalculated to cisplatin equivalent. The determined cisplatin concentration was linearly dependent on applied cisplatin concentration with coefficient of determination R^2^ = 0.98 (Figure 2E) and the determined incorporated cisplatin amount was in the range of 44% – 69% of applied cisplatin concentrations (Figure 2E inset).

**Figure 3.**
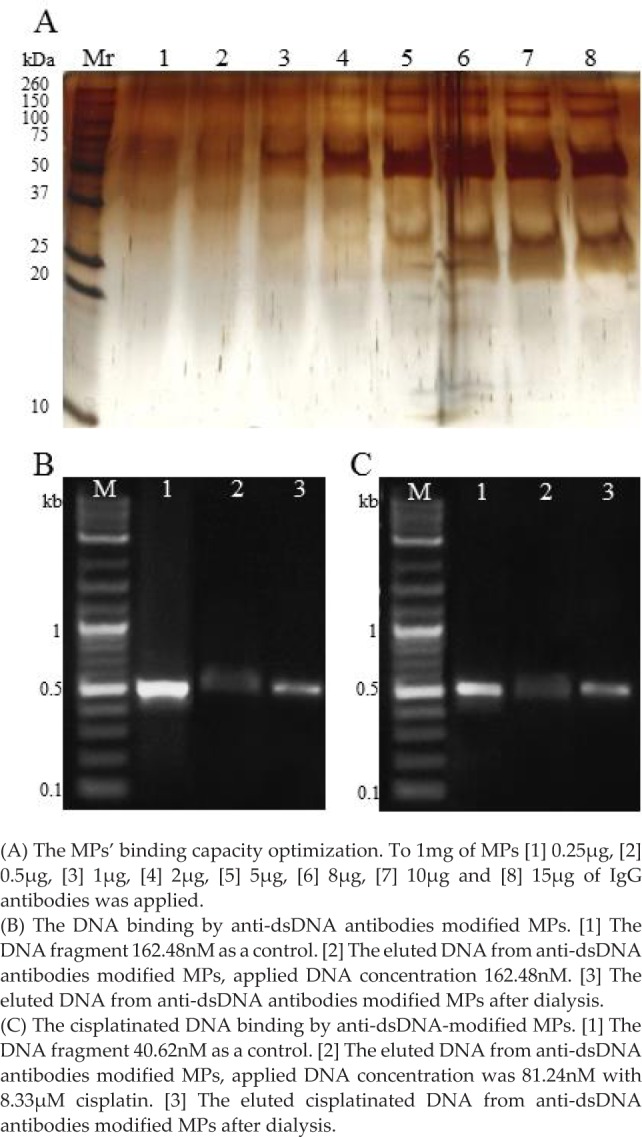
The characterization of MPs coated with antibodies evaluated by gel electrophoresis.

To ensure the complete saturation of the protein-G-paramagnetic particles surface, the capacity of the beads was tested. The amount of antibodies released from the beads' surface eluted by the effect of detergents and reducing agents present in the reducing SDS-PAGE sample buffer was determined by silver staining. The capacity of the beads was assessed as 10μg of antibodies permg of the beads from the increase in band intensities with size of 150, 50 and 30 kDa (corresponding to IgG whole molecule, heavy and light chains, respectively) (Figure 3A). Usability of the antibody-modified beads for DNA extraction from the solution was tested by agarose electrophoresis. In Figure 3B the result of immunoextraction of dsDNA by beads modified with anti-dsDNA antibody is shown. When the band intensities before and after extraction were compared, it is obvious that when using 162.5nM concentration of the PCR product (0.38 ng/mg of MPs), 37% of the original DNA amount was eluted (92.5 ng). To test the extraction of cisplatinated DNA, the beads modified with anti-dsDNA antibody were used, Figure 3C. When comparing the bands' intensity before and after extraction, it is obvious that when using 40.62nM DNA interacted with 8.3μM cisplatin (62.5 ng), 86% of the original DNA amount was eluted (53.75 ng).

**Figure 4.**
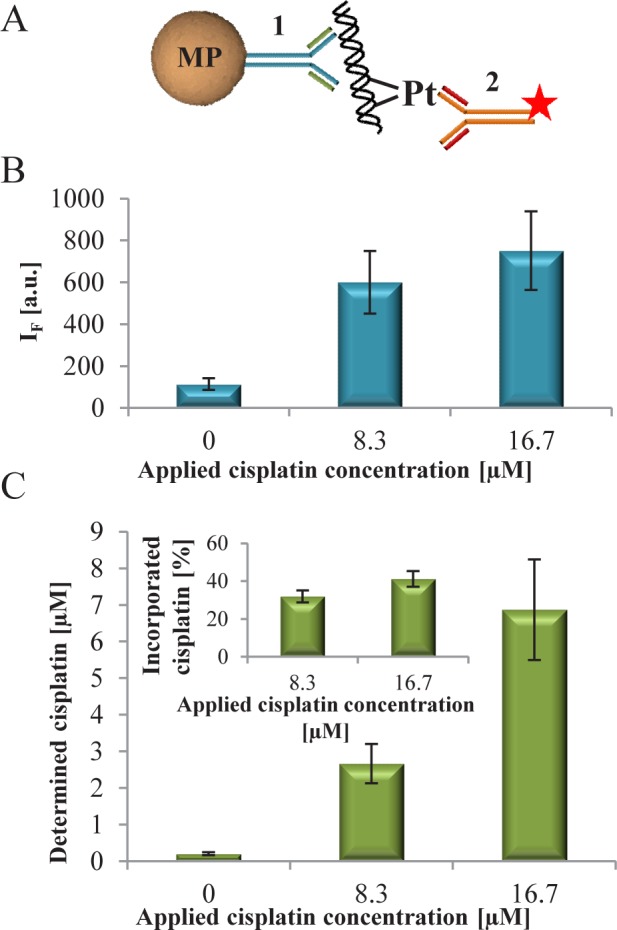
(A) The scheme of sandwich immunoassay on MPs where [1] the anti-dsDNA antibody and [2] anti-cisplatin-modified DNA antibody labelled by QDs. (B) The fluorescence intensity of QDs coupled to anti-cisplatin-modified DNA antibodies, which labelled the cisplatinated DNA bound to MPs modified by anti-dsDNA antibodies. (C) The cisplatin concentration determined by LA-ICP-MS after elution from MPs. Inset: calculated percentage of cisplatin incorporated in DNA that was immunoseparated.

On the basis of the obtained results, a bead-based sensor for cisplatinated DNA was proposed. The construction of a sensor composed from sandwich construction bead – anti-dsDNA antibody – cisplatinated DNA – QD-labelled anti-cisplatin-modified DNA antibody (Figure 4A). The process of antibodies labelling by QDs was adopted from [22]. 40.62nM (125 ng) lambda DNA was interacted with 0, 8.3 and 16.7μM cisplatin and consequently used for testing of the sensor. Compared to DNA without cisplatin, at all applied cisplatin concentration a 5.3- or 6.6-fold increase in fluorescence was observed (Figure 4B). The fluorescence signal of the sensor without DNA was undetectable.

The concentration of Pt in eluted DNA was determined by LA-ICP-MS and recalculated to cisplatin equivalent (Figure 4C). When compared with the applied and eluted cisplatin, in the eluted DNA solution 32% of applied cisplatin was present for 8.3μM cisplatin and 42% for 16.7μM cisplatin. This confirms that the recorded fluorescent signal is caused by the presence of cisplatin-modified DNA.

The proposed procedure will be tested for quantification of DNA adducts in cisplatin-treated tumour cells [27]. After using different antibodies, which recognize either particular type of DNA damage (DNA breaks, doxorubicin modifications, short DNA fragments, etc.), this procedure can be easily modified for concrete application, for example, determination of free DNA in autoimmune diseases, such as systemic lupus erythematosus or circulating tumour DNA [28]. In the 1970s, tumour imaging utilizing radioiodinated DNA for tumour imaging was published [29]. After cisplatin therapy, the proposed sensor could be usable for theranostics (double imaging using QDs and paramagnetic particles [30]) and hyperthermia (paramagnetic particles) [31].

Among the analytical approaches currently used for measuring DNA adducts, we can include ^32^P-post-labeling, immunoassay using antibodies against modified DNA, conventional mass spectrometry (MS) and accelerator MS [32]. Immunoassays are powerful tools for the analysis of DNA adducts. They have provided sufficient sensitivity and selectivity for quantification of clinically relevant low levels of DNA modifications [33]. However, immunoassays do not provide structural information about adducts. The high sensitivity of the ^32^P-post-labeling is most frequently used for DNA-adduct detection [34]. However, it suffers from several drawbacks, including the need for reference standards and the inability to characterize unknown adducts. Moreover, it is susceptible to false positives or false negatives, it utilizes radioactivity and numerous sample pretreatment steps have to be conducted to ensure that adducts are well purified and isolated from nonadducted DNA fragments. Thus the ^32^P-post-labeling procedure is still very laborious; the time-span for analysis is of the order of days. One important limitation of this method is the variable yield of the different enzymatic reactions (necessary for preparation of ^32^P-labeled species), which complicates absolute quantitative determination of DNA adducts [32]. Significant advances have been made in developing MS-based approaches with improved sensitivity and versatility, to provide new insights and more conclusive information due to the high specificity inherent in the MS technique. It is therefore now possible to characterize and to determine platinated DNA adducts by MS-based methodologies with sensitivity comparable to that of ^32^P-post-labeling or immunoassays [35].

Paramagnetic particle-based immunoassay requires less time and lower sample amount to perform the analysis and labelling with nanoparticles offers the possibility of multianalyte determination [23, 36].

## 4. Conclusions

We suggested an easy magnetic separation-based assay for DNA quantification, which is usable for quantification of DNA lesions in cells treated with cytostatics. Using antibodies, which recognize either particular type of DNA damage (DNA breaks, doxorubicin modifications, short DNA fragments, etc.), allows easy modification of this procedure for concrete application. After cisplatin therapy, the proposed sensor could be usable for theranostics (double imaging using QDs and paramagnetic particles) and hyperthermia (paramagnetic particles).
